# Human cytomegalovirus infection is correlated with enhanced cyclooxygenase-2 and 5-lipoxygenase protein expression in breast cancer

**DOI:** 10.1007/s00432-019-02946-8

**Published:** 2019-06-15

**Authors:** Helena Costa, Joel Touma, Belghis Davoudi, Melinda Benard, Torill Sauer, Jürgen Geisler, Katja Vetvik, Afsar Rahbar, Cecilia Söderberg-Naucler

**Affiliations:** 1 Department of Medicine, Solna, Microbial Pathogens Unit, BioClinicum at Karolinska Institute, Solna, Sweden; 2 Division of Neurology, Karolinska University Hospital, Stockholm, Sweden; 3 Department of Breast and Endocrine Surgery, Akershus University Hospital (AHUS), Lørenskog, Norway; 4 INSERM, UMR 1043, Hospital Purpan, Paul Sabatier University, 31024 Toulouse, France; 5 Neonatal Unit, Children’s Hospital, 31059 Toulouse, France; 6 Institute of Clinical Medicine, University of Oslo, Oslo, Norway; 7 Department of Pathology, Akershus University Hospital, Lørenskog, Norway; 8 Department of Oncology, Akershus University Hospital, Lørenskog, Norway

**Keywords:** Human cytomegalovirus, Breast cancer, 5-LO, COX-2, MCF-7, MDA-MB-231

## Abstract

**Purpose:**

While enhanced expression of cyclooxygenase-2 (COX-2) and 5-lipoxygenase (5-LO) and their derived metabolites is associated with breast cancer (BC) risk, the precise link between BC carcinogenesis and enhanced inflammatory activity remains to be clarified. Human Cytomegalovirus (HCMV) may induce expression of COX-2 and 5-LO and is frequently found in breast cancer biopsies. Thus, we investigated whether there is an association between HCMV proteins and expression of COX-2 and 5-LO in human BC tissue and BC cell lines.

**Materials and methods:**

Paraffin embedded biopsies obtained from 49 patients with breast cancer and 26 tissue samples from adjacent, benign breast tissues were retrospectively examined for HCMV-immediate early (IE), HCMV-Late (LA), COX-2, and 5-LO proteins by immunohistochemistry. In vitro, uninfected and HCMV-infected BC cell lines were examined for COX-2 and 5-LO transcripts and proteins by PCR and flow cytometry.

**Results:**

Extensive expression of COX-2, 5-LO and HCMV-IE proteins were preferentially detected in BC samples. We found a statistically significant concordant correlation between extensive HCMV-IE and COX-2 (*P* < 0.0001) as well as with HCMV-IE and 5-LO (*P *= 0.0003) in infiltrating BC. In vitro, HCMV infection induced COX-2 and 5-LO transcripts and COX-2 proteins in MCF-7 cells (*P *=0.008, *P *=0.018, respectively). In MDA-MB-231 cells that already had high base line levels of COX-2 expression, HCMV induced both COX-2 and 5-LO proteins but not transcripts.

**Conclusion:**

Our findings demonstrate a significant correlation between extensive HCMV-IE protein expression and overexpression of COX-2 and 5-LO in human breast cancer.

**Electronic supplementary material:**

The online version of this article (10.1007/s00432-019-02946-8) contains supplementary material, which is available to authorized users.

## Introduction

Breast cancer (BC) is the most common cancer in women worldwide and although implementation of novel therapies and early detection has improved quality of life and decreased mortality rates for some BC subtypes, the overall incidence of BC continues to increase (Jemal et al. 2011). Of high concern, the overall survival has not improved significantly albeit new therapies for patients with metastatic BC have been introduced during the past decades (https://www.kreftregisteret.no/globalassets/publikasjoner-og-rapporter/cancer-in-norway/cancer_in_norway_2009.pdf), suggesting that alternative treatment options are urgently needed. Somatic mutations in breast cancer-associated genes are today considered to be one of the main causes and drivers of BC. In addition, epigenetic and genetic alterations in key breast cancer genes such as BRCA-1/2 and their downstream regulation of DNA repair mechanisms, or in genes with impact on epithelial cell proliferation, differentiation and migration are suggested to promote breast cancer carcinogenesis (Petrucelli et al. 1993). Emerging evidence also highlights a role of the tumor microenvironment to induce pre-malignancies and promote tumor progression. Stroma cells like specialized mesenchymal cells and myo-fibroblasts, endothelial cells and infiltrating leukocytes with capacity to produce inflammatory factors may promote breast cancer carcinogenesis and affect tumor progression and metastasis formation (Balkwill et al. 2005; Coussens and Werb 2002; de Visser et al. 2006). Inflammatory cells present in the tumor microenvironment support tumor growth and enhanced malignancy, by releasing cytokines, chemokines and growth factors. Their increased production of reactive oxygen species also further enhance inflammation, oxidative DNA damage and impair DNA repair mechanisms (Coussens and Werb 2002). Therefore, chronic inflammation is considered a risk factor for breast cancer development, and added inflammation to their revised version of the Hallmarks of Cancer (Hanahan and Winberg 2011).

Eicosanoids are potent inflammatory mediators that are produced from arachidonic acid by cyclooxygenases (COXs) and lipoxygenase (LO). Enhanced inflammation via COX-2 and 5-LO promotes tumorigenesis (Chen and Smyth 2011) and is associated with poor prognosis of several cancer forms (Dreyling et al. 1986; Hennig et al. 2002; Steele et al. 1999). Over-expression of COX-2 was observed in 40% of patients with invasive breast carcinoma and correlated with poor prognosis (Denkert et al. 2003; Howe 2007). Selective COX-2 inhibitors reduce the risk of breast cancer, suppress breast cancer cell migration and invasion, and exhibit strong anti-neoplastic effects in animal models (Wang and DuBois 2010; Harris et al. 2006, 2014). COX-2 inhibitors are, therefore, currently evaluated as potential new cancer therapies, especially in epithelial-derived malignancies. A randomised phase II study of 111 postmenopausal women with advanced breast cancer indicated a trend towards a longer duration of clinical benefit in the combination arm (median, 96.6 weeks vs 49 weeks) compared to the exemestane monotherapy arm (Dirix et al. 2008). Inhibition of 5-LO activity in several breast cancer cell lines also resulted in growth inhibition and enhanced apoptosis, but 5-LO inhibitors have not yet been evaluated in clinical trials in breast cancer patients (Avis et al. 2001).

Human cytomegalovirus (HCMV) is a ubiquitous herpesvirus that is proposed to be highly prevalent in different cancer forms including breast (Harkins et al. 2010; Taher et al. 2013, 2014), colon (Dimberg et al. 2013; Harkins et al. 2002; Tafvizi and Fard 2014) and prostate cancer (Samanta et al. 2003), glioblastoma multiforme (Cobbs et al. 2002; Lucas et al. 2011; Rahbar et al. 2015), medulloblastoma (Baryawno et al. 2011) and neuroblastoma (Wolmer-Solberg et al. 2013). HCMV is also present in sentinel lymph node metastases of breast cancer and in brain metastases originating from breast and colon cancer, while healthy tissues surrounding HCMV positive primary tumors are generally HCMV negative. In breast cancer, HCMV was detected in 92% of primary tumors, in 94% of sentinel lymph nodes, and in 99% of brain metastases originating from breast cancer (Taher et al. 2013, 2014). These observations suggest a concerning association between HCMV and malignant cell growth. However, it remains to be clarified whether these findings represent an epiphenomenon or indicate that HCMV infections induce tumor-promoting mechanisms of relevance for local tumor progression.

A close link between HCMV infections and inflammation has been established in the literature. Thus, latent HCMV can be reactivated by inflammation and the virus further promotes inflammation by inducing expression of both COX-2 and 5-LO (Benard et al. 2014; Qiu et al. 2008; Hooks et al. 2006). Results from a mouse model based on virus-induced adenocarcinoma (Bongers et al. 2010), confirmed production of inflammatory factors such as IL-10, TGF-β, IL-1β, IL-8, IL-6, MIP-1α, MIP-1β, and RANTES (Bodaghi et al. 1998; Kotenko et al. 2000; Reeves et al. 2005; Söderberg-Nauclér et al. 1997) to be involved. We earlier showed that COX-2 is almost exclusively expressed by HCMV-infected cells in medulloblastoma and that COX-2 inhibition acts to inhibit HCMV, which led to decreased tumor growth in an animal model (Baryawno et al. 2011; Schroer and Shenk 2008). In addition to inducing inflammation, HCMV may directly modulate tumor cells by affecting intracellular pathways involved in cell cycle regulation, epigenetic regulation of gene expression, cellular invasion, angiogenesis, immune evasion, and apoptosis (Cinatl et al. 2004a, b; Slinger 2010; Vossen et al. 2002), all highly relevant in tumor biology.

The main objective of this study was to investigate a potential association between HCMV infection and simultaneous expression of COX-2 and 5-LO in BC. We examined the expression of HCMV proteins, COX-2 and 5-LO, in tissue samples obtained from BC and in adjacent normal breast tissues and investigated whether the activity level of HCMV was associated with inflammatory markers and impaired clinical outcome. In additional in vitro experiments, we assessed whether HCMV could affect the expression of 5-LO and COX-2 in well-established BC cell lines.

## Materials and methods

### Study design

Paraffin-embedded tissue specimens of infiltrating BC (*n* = 75) and adjacent normal breast tissue (*n* = 26) were retrospectively obtained from 49 patients who underwent surgery at Akershus University Hospital, Oslo, Norway during 2011. Clinical data (Table 1) were provided by the Departments of Oncology and Pathology at Akershus University Hospital (AHUS). All diagnoses were re-confirmed by an experienced BC pathologist (T.S.) at AHUS. The median age at surgery was 58.7 years. Most patients underwent mastectomy (61%), while 35% had breast-conserving surgery, and 4% bilateral surgery. All patients received standard adjuvant treatment according to the Norwegian guidelines approved at the time of surgery (Table 1); (http://www.nbcg.no).

**Table 1:** Patients characteristic

	*n* (%)
Patient characteristics	
Patients	49
Median age (years)	49
Median age at surgery (years)	58.7
Type of surgery	
Breast conserving	17 (35)
Ablation	30 (61)
Bilateral	2 (4)
Histology	
Infiltrating ductal cancer	41 (84)
Infiltrating lobular cancer	4 (8)
Medullar cancer	0 (0)
Multiple cancer types	1 (2)
Mucinous subtype	3 (6)
Grades (*n* = 48)	
I	6 (13)
II	17 (35)
III	25 (52)
T stadium (*n* = 49)	
T1	25 (51)
T2	24 (49)
N stadium (*n* = 49)	
N0	29 (59.2)
N1	11 (22.4)
N2	7 (14.3)
N3	2 (4.1)
ER expression	
Positive (≥ 1%)	35 (71)
Negative	14 (29)
PGR expression	
Positive (≥ 10%)	23 (47)
Negative	26 (53)
HER2 expression	
Positive (IHC3 + or FISH +)	8 (16)
Negative	41 (84)
Ki-67 labeling index (*n* = 42)	
< 1%	1 (2.4)
> 1–30%	22 (52.4)
> 31–100%	19 (45.2)
Menopause (*n* = 18)	
Premenopause	5 (28)
Postmenopause	13 (72)
Neoadjuvant treatment	
Yes	1 (2)
No	48 (98)
Adjuvant chemotherapy	
No chemotherapy	19 (39)
FEC60 × 6	2 (4)
FEC60 × 4 followed by taxanes (every 3 weeks)	19 (39)
FEC100 × 4 followed by taxanes (every 3 weeks)	9 (18)
Adjuvant endocrine treatment	
None	19 (38.8)
Tamoxifen (5 years)	15 (30.6)
Aromatase inhibitor (5 years)	15 (30.6)
Adjuvant radiation	
No	17 (35)
Yes	32 (65)
Adjuvant anti-HER2 treatment	
No	41 (84)
Yes	8 (16)
Relapse	
No	45 (92)
Yes	4 (8)
Loco regional relapse	
No	49 (100)
Distant metastasis	
No	44 (90)
Yes	5 (10)
Type of distant metastasis (*n* = 5)	
Lymph nodes	2 (40)
Brain	2 (40)
Multiple metastasis	1 (20)
Alive	
Death	4 (8)
Alive	45 (92)

*T stadium* tumor size and extension into neighboring breast tissue, *N stadium* lymph node involvement

### Immunohistochemistry

Tissue microarrays were created, and all tissues were sectioned (4 µm) and analyzed by immunohistochemical techniques optimized in our laboratory. Detection of HCMV proteins was done as described previously with only minor modifications (Taher et al. 2013). Tissue specimens were deparaffinized in xylene (Sigma Aldrich), rehydrated in an ethanol (Apoteket Farmaci), and washed in Tris-buffered saline (TBS) containing Triton X-100 (Substrate Department, Karolinska University Hospital). Antigen retrieval and unmasking was done by heating the tissues in DIVA decloaker buffer (Histolab), pH 6.2, in a pressure cooker (BioCARE) for 15 min. Endogenous peroxidase activity was blocked with peroxidase 1 (Histolab) for 5 min, and nonspecific binding was blocked with Sniper (Histolab) for 16 min at room temperature. The tissue sections were then incubated with antibodies against HCMV-IE and HCMV-LA (IgG2a, Merck), COX-2 (CellSignaling), 5-LO (Abcam), and cytokeratins 5, 6, 8, 17, and 19 (IgG1, Dako). An antibody against cytokeratin 20 (IgG2a, Chemicon International) and rabbit IgG (Biocare Medical) served as negative controls. Paraffin-embedded tissue section from HCMV-infected placenta was used as positive control and from HCMV negative breast cancer patient as negative control for IHC.

During the staining procedure a few of the tissue sections were lost.

HCMV, COX-2, and 5-LO staining was evaluated as described previously (Taher et al. 2013), and according to the estimated percentage of cells expressing HCMV or COX-2 or 5-LO proteins: negative (0%), grade 1 (< 25%), grade 2 (≥ 25–50%), grade 3 (≥ 50–75%), and grade 4 (≥ 75%). To ensure a sufficient number of cases in each category for statistical analysis, tumors were considered as HCMV-negative or as having focal HCMV infection (< 50% positive cells) or extensive infection (≥ 50% positive cells). Immunohistochemical (IHC) staining for HCMV was evaluated and graded by a senior scientist (A.R.) without access to the clinical records at Karolinska Institutet, Stockholm, while IHC staining for Ki-67 was performed and evaluated at the Department of Pathology, Akershus University Hospital.

### Cell lines and virus

Breast cancer cell lines MCF-7 (ER/PR/ positive but HER2 negative) and MDA-MB-231 (ER/PR/HER-2 negative), both from ATCC, were cultured in RPMI 1640 medium supplemented with 10% foetal bovine serum (FBS), 100 U/ml of penicillin and 100 µg/ml of streptomycin and maintained in a 37-C incubator with 5% CO_2_. Viral stocks of HCMV VR1814 strain were prepared through virus propagation in human umbilical vein endothelial cells (HUVEC) at low passage and ultracentrifugation of supernatants as described earlier (Frascaroli and Sinzger 2014).

### RNA extraction and quantitative real-time PCR (qPCR)

MCF-7 and MDA-MB-231 cells were infected with HCMV VR1814 at multiplicity of infection (MOI) of 5 and were collected at different times post-infection. RNA was extracted from lysed cells using RNeasy Mini Kit (Qiagen) according to manufacturer’s instructions and cDNA was synthesized using random primers and the high-capacity cDNA reverse transcription kit (Applied Biosystems). Gene expression levels were quantified by real-time PCR using TaqMan Fast Universal PCR Master Mix (Life Technologies) and the following specific TaqMan probes: COX-2 (PTGS2, assay ID Hs00153133_m1), 5-LO (ALOX5, assay ID Hs00167536_m1), HCMV IE, and human β2-microglobulin (B2M, assay ID, Hs00984230_m1) (Life Technologies). The PCR was performed using a 7900HT Fast Real-Time PCR system (Applied Biosystems). The endogenous control B2M was used for normalization and relative expression was determined by the 2^−ΔΔCt^ method. (Three separate experiments were performed).

### Flow cytometric analysis (FACS)

MCF-7 and MDA-MB-231 cells non-infected or infected with HCMV MOI of 5 were fixed at 6 days post infection (dpi). Cells were treated in Fix &Perm Medium A and B according to the supplier`s instruction (Molecular Probes) and co-stained with primary antibodies HCMV IE (Merck) and COX-2 (CellSignaling) or 5-LO (Abcam) diluted in phosphate-buffered saline (PBS) containing 1% BSA (Sigma) for 45 min at 4 °C. After washing with PBS, cells were incubated with secondary Alexa Fluor 633 goat anti-mouse antibodies (Life Technologies) and Alexa Fluor 488 anti-rabbit antibodies (Invitrogen). Rabbit IgG (Biocare Medical) and mouse IgG2a (Dako) served as negative controls. Acquisition was performed using Flow cytometry (FACS) and data were analyzed by Summit 4.3 software. (Three separate experiments were performed).

### Statistical analysis

All analyses were done with GraphPad Prism version 6; *P* < 0.05 was considered statistically significant. Nonparametric Pearson correlation assay was used to assess statistical significance of correlation between different grades of HCMV-IE, COX-2, and 5-LO in BC tissues. Two-tailed Student *t* test was used for analysis of in vitro data, presented as mean standard deviation.

## Results

### High prevalence of HCMV-IE, COX-2, and 5-LO proteins in BC, but not in adjacent non-malignant breast tissues

**Fig. 1:**
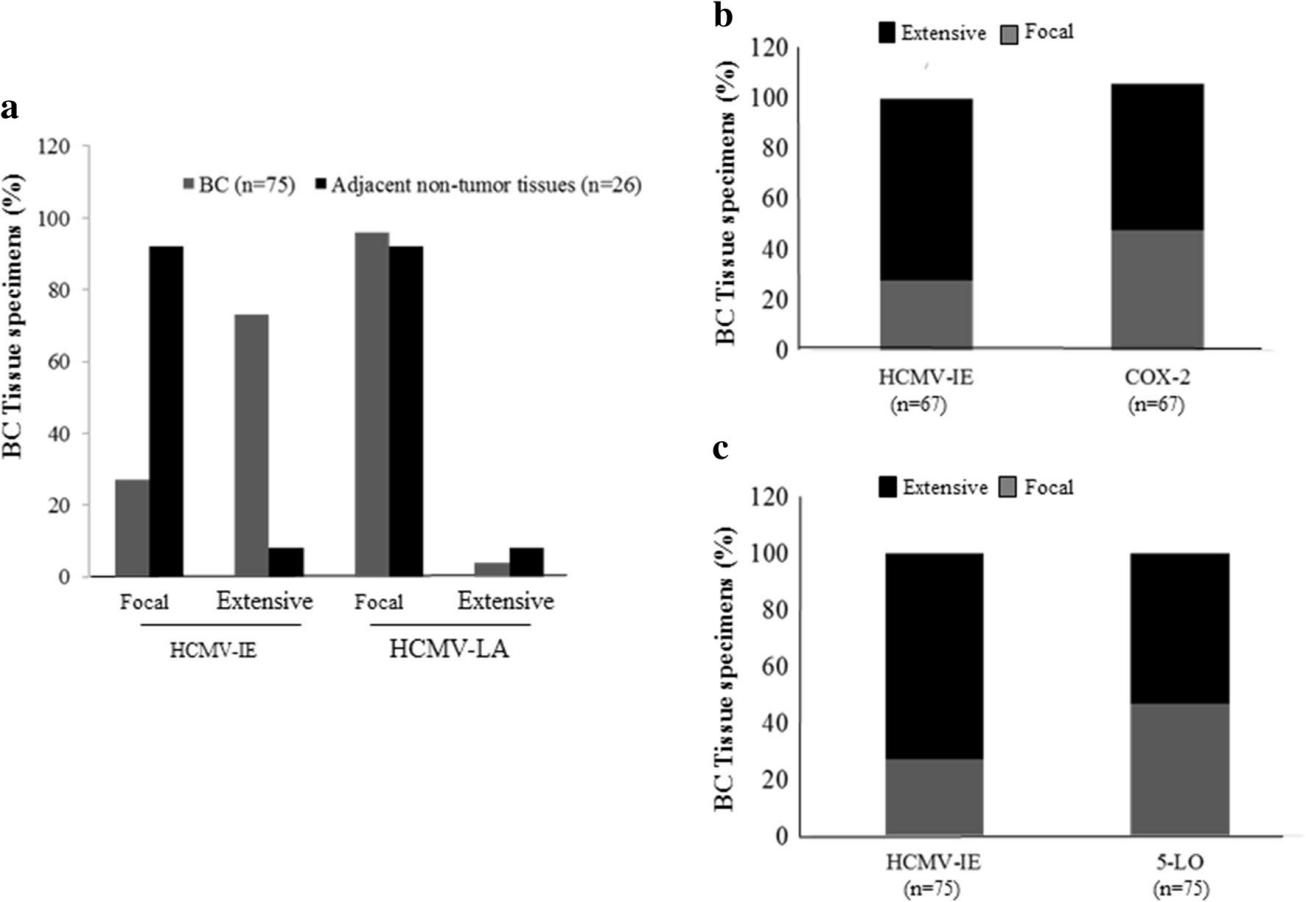
Detection of HCMV-IE and HCMV-LA in infiltrated BC and adjacent normal breast tissue by immunohistochemical staining (IHC). Extensive HCMV-IE but not HCMV-LA was frequently detected in infiltrated BC (**a**). Extensive HCMV-IE and COX-2 were detected in majority of infiltrated BC (**b**, **c**)

**Fig. 2:**
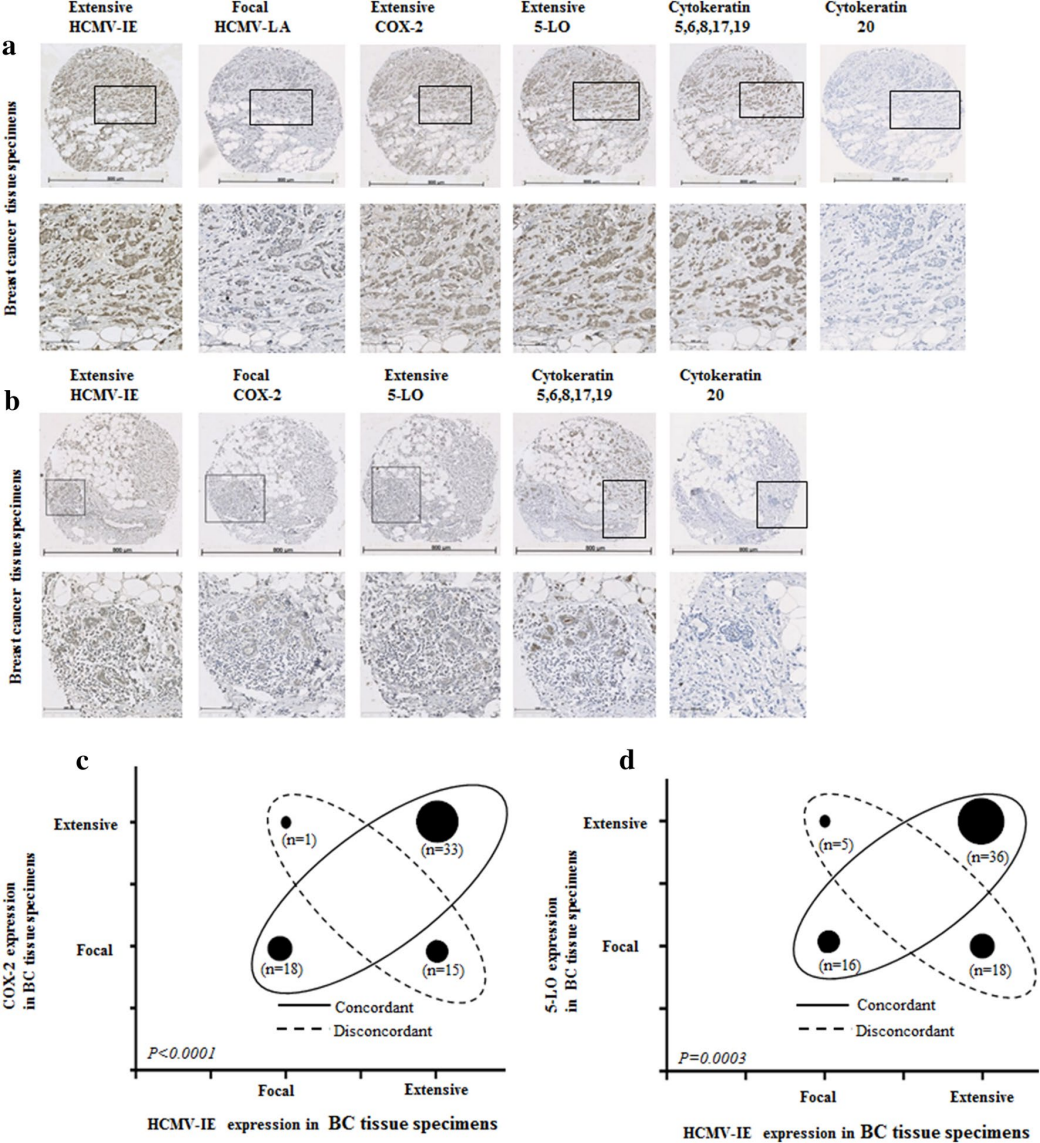
Concordant expression levels of HCMV-IE and COX2/5-LO in BC tissue specimens (**a**, **b**). Images show extensive expression of HCMV-IE, COX-2 and 5-LO in the same BC tissue specimens (**a**). Images show focal expression of HCMV-IE, COX-2 and 5-LO in the same BC tissue specimens (**b**). Lower panel present magnified images from upper panel. A significant correlation was detected between concordant expression levels of HCMV-IE, COX-2 and HCMV-IE, 5-LO (**c**, **d**)

**Fig. 3:**
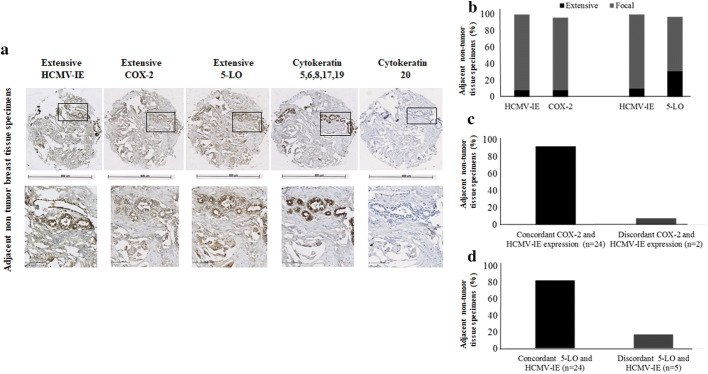
Correlation between concordant expression of HCMV-IE, COX-2 and 5-LO in adjacent non-tumor breast tissue specimens. Images show extensive expression of HCMV-IE, COX-2 and 5-LO in the same adjacent non-tumor tissue specimens (**a**). Lower panel present magnified images from upper panel. Majority of adjacent non-tumor breast tissues have focal expression of HCMV-IE, COX-2 and 5-LO (**b**). Concordant expression of HCMV-IE and COX-2/5-LO were detected in majority of adjacent non-tumor breast tissues (**c**, **d**)

Expression of HCMV-IE, HCMV-LA, COX-2, and 5-LO proteins was analyzed in available tissue sections by immunohistochemistry. HCMV IE protein was detected at different levels in breast cancer (BC) tissue specimens from all 49 patients and HCMV-LA protein was found in 22% (11/49) of BC patients. HCMV IE protein expression was extensive (defined as > 50% positive cells) in 73% (55/75) of BC tissue specimens, but only in 8% (2/26) of adjacent breast tissue specimens (Figs. 1, 2, 3). This cohort of breast cancer patients was previously investigated for HCMV protein expression and association with estrogen receptor-alpha (ER) and progesterone receptor (PGR) expression and the results were reported earlier (Rahbar et al. 2017). Most of tumor specimens exhibited low expression of HCMV LA proteins; extensive HCMV-LA expression was only present in 3 of 75 (4%) of BC specimens, and in none of adjacent breast tissue specimens (Figs. 2, 3). COX-2 protein expression was extensive in 58% (48/67) of BC specimens and in 8% (2/26) of adjacent non-tumor breast specimens (Figs. 1, 2, 3). 5-LO protein expression was extensive in 53% (40/75) of BC specimens and in 31% (9/29) of adjacent non-tumor breast specimens (Figs. 1, 2, 3). Expression of HCMV-IE protein was detected in HCMV positive placenta tissue section, but not in HCMV negative breast tumor tissue section by immunohistochemistry (Supplemental Fig. 1A-B).

### Significant positive correlation between HCMV-IE protein, COX-2 and 5-LO expression

We further assessed whether there was a correlation between HCMV protein expression, COX-2, and 5-LO expression. A positive correlation was found between extensive HCMV-IE protein expression and extensive COX-2 or 5-LO protein expression in breast cancer tissue specimens (49% and 48%, respectively) (Fig. 2a–d). Significantly higher number of BC tissues had equal levels of both HCMV-IE/COX-2 and HCMV-IE/5-LO protein expression (concordant); 76% (51/67, *P *< 0.0001) and 69% (52/75, *P *= 0.0003), respectively (Fig. 2a–d). In adjacent non-tumor breast tissue, we instead found concordant levels of focal HCMV-IE/COX-2 and HCMV-IE/5-LO in 92% (24/26), and 83% (24/29) of the samples, respectively (Fig. 3a–d). Extensive expression of HCMV-IE and COX-2/5-LO was located in the same areas within the tissue sections. But in case of focal expression, these proteins were mainly expressed not only in the inflammatory cells but also in some tumor cells and in the cells of vessel walls.

Clinical data including Ki-67 labeling index, nodal status, and tumor stadium for BC patients are shown in Table 1. We found no association between HCMV-IE, COX-2 and 5-LO and lymph node involvement, tumor size or Ki-67 index (Supplemental Figs. 2, 3). At the time of our investigation, 92% (45/49) of BC patients were alive and median overall survival was 4.4 years. We also found no association between extensive HCMV infection and these inflammatory markers with patient outcome, but as only few patients in this cohort had relapsed or died, these results are still premature and somewhat uncertain.

### HCMV induces COX-2 and 5-LO transcript levels in MCF-7 but not in MDA-MB-231 breast cancer cell lines

**Fig. 4:**
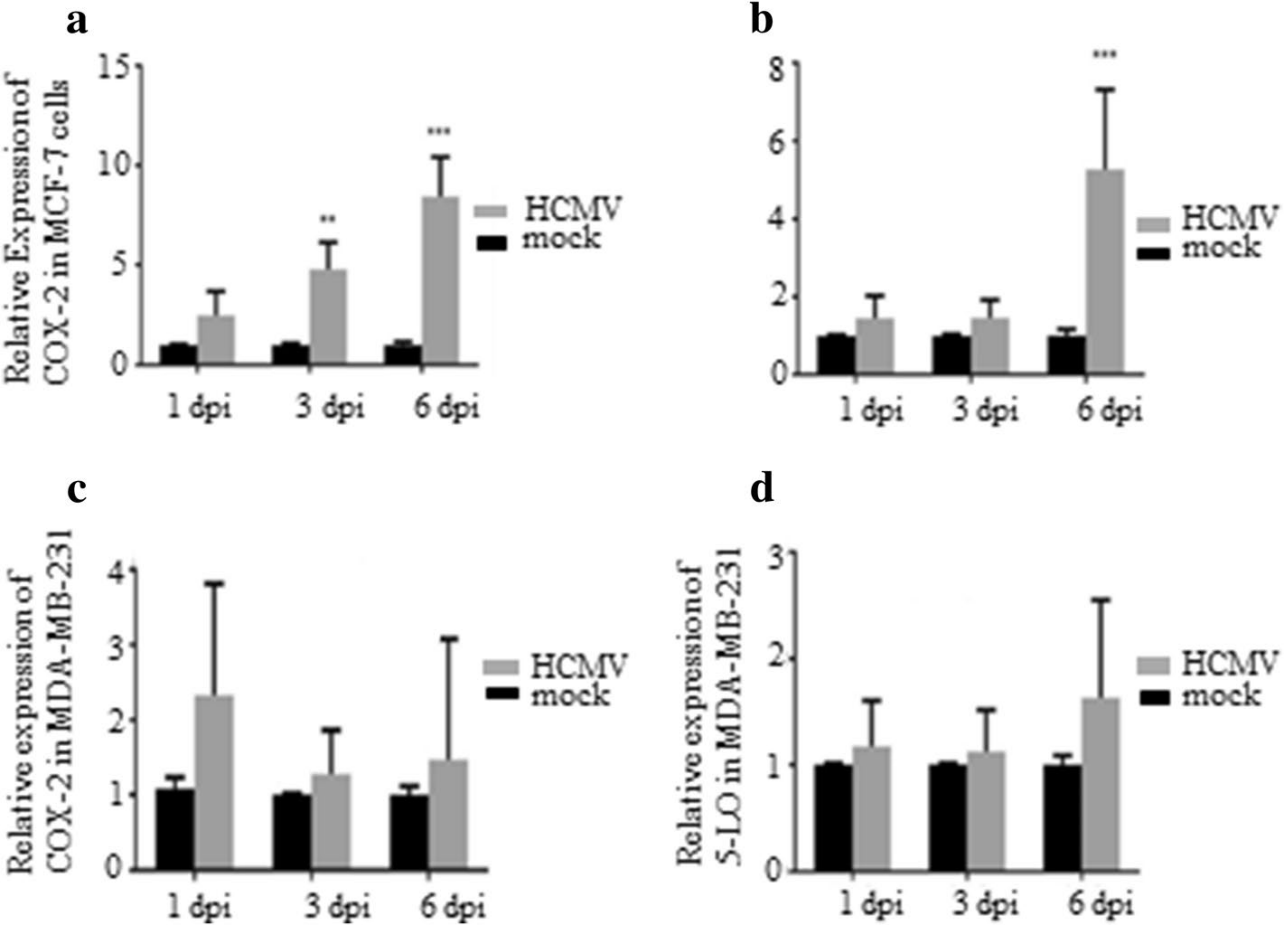
Significant increase of COX-2 and 5-LO transcripts in HCMV infected MCF-7 but not in MDA-MB-231 breast cancer cell lines. Relative expression of COX-2 and 5-LO in MCF-7 (**a**, **b**, respectively) or MDA-MB-231 (**c**, **d**, respectively) infected with HCMV compared to non-infected cells was determined by qPCR at 1, 3 and 6 dpi. Data is presented as mean ± SD. Statistical significance is indicated as **P < 0.01, ***P < 0.001

**Fig. 5:**
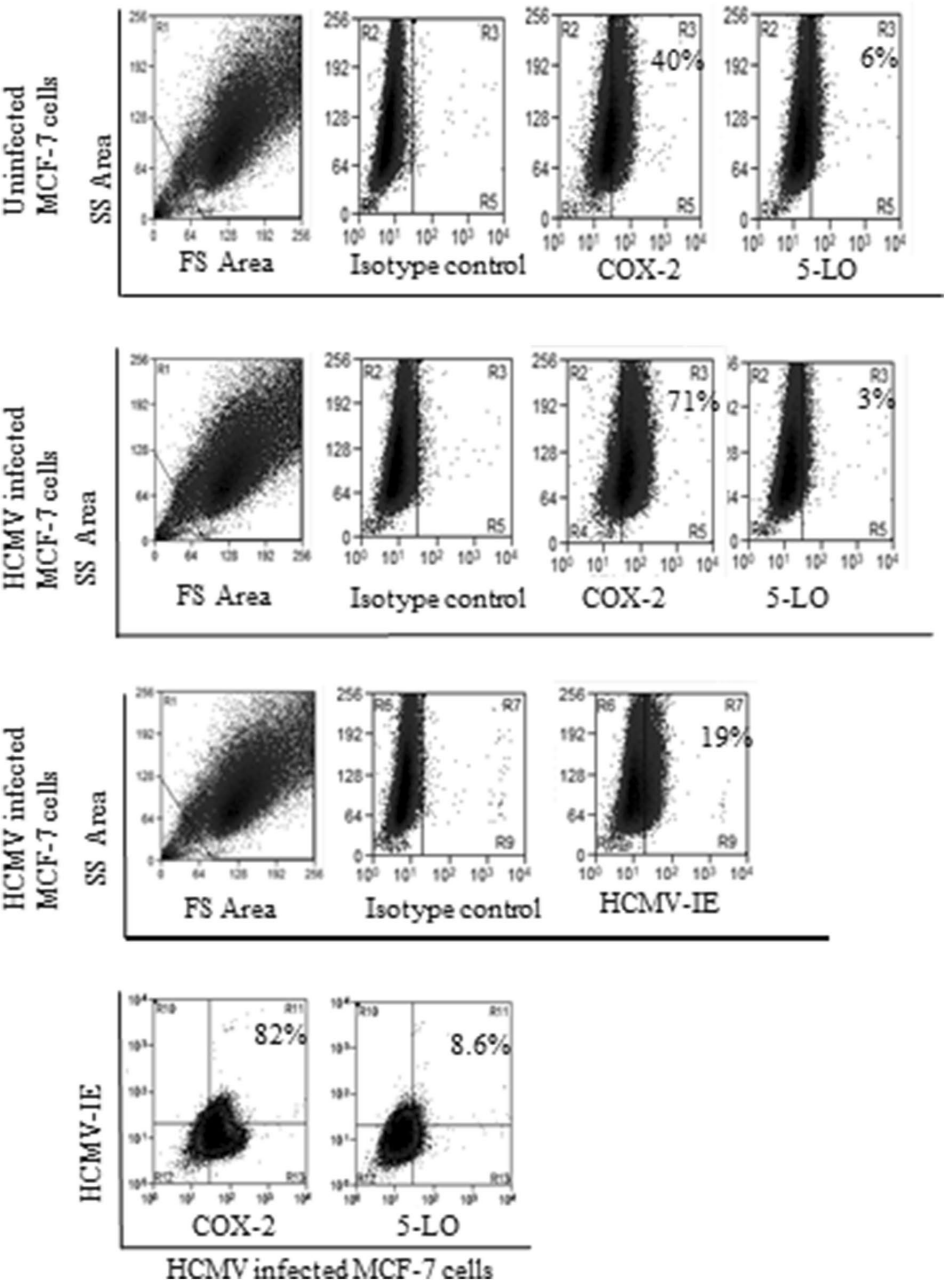
The number of cells expressing COX-2 but not 5-LO increased in HCMV infected MCF-7 cell populations compared with uninfected cells analyzed by using FACS. Eighty-two percent of HCMV infected MCF-7 cells express COX-2 protein and only 8.6% of the infected cells expressed 5-LO

**Fig. 6:**
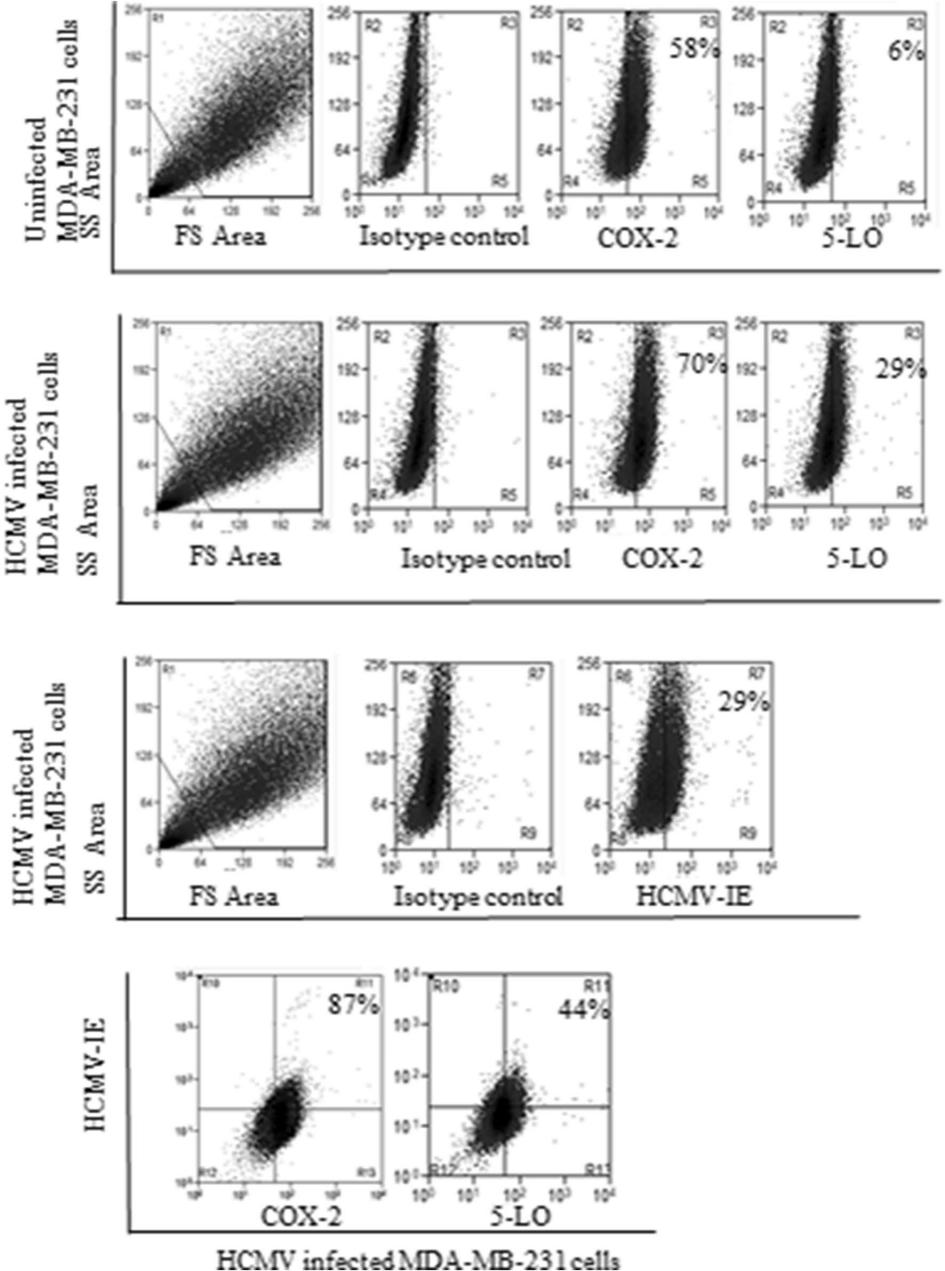
The number of cells expressing COX-2 and 5-LO increased in HCMV infected MDA-MB-231cell populations compared with uninfected cells analyzed by using FACS. Eighty-seven and 44% of HCMV infected MDA-MB-231 expressed COX-2 and 5-LO, respectively

To assess whether HCMV can induce COX-2 and 5-LO expression in breast cancer cell lines, we infected MCF-7 and MDA-MB-231 with HCMV strain VR1814 at MOI of 5 and analyzed transcript levels of COX-2 and 5-LO by qPCR at 1, 3, and 6 dpi. In MCF-7 cells, HCMV infection resulted in a significantly induced COX-2 transcript expression at 3 and 6 dpi (*P *= 0.015 and *P *=0.008, respectively), and 5-LO at 6 dpi (*P *= 0.018) (Fig. 4a, b). However, HCMV infection had no effect on COX-2 or 5-LO transcript levels in MDA-MB-231 cells (Fig. 4c, d) as assessed with qPCR. Uninfected and HCMV-infected BC cell lines MCF-7 and MDA-MB-231 at 6 dpi were further analyzed for protein expression of COX-2 and 5-LO by FACS. Base line COX-2 protein levels were higher in MDA-MB-231 cells compared with MCF-7 cells (Figs. 5, 6). COX-2 was expressed in 58% and 40% of MDA-MB-231 and MCF-7 cells, respectively (Figs. 5, 6). 5-LO was expressed in only 6% of MDA-MB-231 and MCF-7 cells (Figs. 5, 6).

The number of cells expressing COX-2 increased from 58 to 70% in HCMV-infected MDA-MB-231 and from 40 to 71% in MCF-7 cell population. The number of HCMV-induced 5-LO expressing cells increased from 6 to 29% in MDA-MB-231 population, but did not affect 5-LO expression in MCF-7 cells (Figs. 5, 6). Seventy percent of HCMV infected MDA-MB-231 and MCF-7 cells expressed COX-2 (Figs. 5, 6). Twenty-nine percent of HCMV-infected MDA-MB-231 cells, but only 3% of MCF-7 cells expressed 5-LO (Figs. 5, 6).

## Discussion

The presence of HCMV, as well as other herpesviruses in different types of cancers has been studied by several groups with variable results (Cianchi et al. 2006; Rahbar et al. 2017). Several studies have reported a very high prevalence of HCMV in breast cancer and linked the virus activity levels to overall survival (Taher et al. 2013; Tsai et al. 2007). Consistent with these observations, we found HCMV protein expression in all breast cancer tissue specimens examined. Moreover, HCMV IE protein expression was extensive (defined as > 50% positive cells) in 72% of breast cancer samples, but only in 8% of adjacent non tumor tissues. These observations give support for a potential role of HCMV in breast cancer progression (Kumar et al. 2018; Rahbar et al. 2017). As it seems, HCMV infections can provide direct effects on tumor cells by expression of viral proteins that affect tumor biology-relevant mechanisms and by virus-induced inflammation. In fact, HCMV seems to contribute to all established Hallmarks of Cancer (Soroceanu and Cobbs 2011).

In support of this statement, we detected extensive expression of the inflammatory factors COX-2 and 5-LO in infiltrating breast cancer (58%, 53%, respectively), but less pronounced activity of these inflammatory mediators in adjacent non-tumor breast tissues (8% and 31%, respectively). These inflammatory factors were expressed in the tumor and in inflammatory cells. We also found a concordant extensive expression of COX-2, 5-LO, and HCMV-IE in breast cancer, suggesting that HCMV activity on tumor cells may be linked to COX-2 and 5-LO expression. This hypothesis was further supported by in vitro experiments demonstrating virus-induced transcript of both COX-2 and 5-LO and COX-2 protein expression in HCMV-infected MDA-231 and MCF-7 cells. However, we did not observe higher expression of COX-2 or 5-LO transcripts in HCMV-infected MDA-231 cells, which may be explained by the already high expression levels of COX-2 proteins in the more aggressive cell line MDA-231 that was originally established from a triple negative breast cancer (TNBC). Previous studies have reported high expression levels of COX-2 in TNBCs (Chikman et al. 2014; Mosalpuria et al. 2014), and we recently reported that HCMV protein expression was high in TNBC (Rahbar et al. 2017). Our results confirm previous studies demonstrating increased levels of COX-2, 5-LO, and their metabolites prostaglandins and leukotrienes in HCMV-infected cells both in vitro and in vivo (Maussang et al. 2009; Qiu et al. 2008; Hooks et al. 2006; Bongers et al. 2010).

HCMV-induced COX-2 expression is mediated by the constitutively active viral chemokine receptor homologue US28, which promotes inflammation, angiogenesis, and tumor formation (Maussang et al. 2009). US28 promotes angiogenesis and tumor formation via COX-2 induced production of IL-6, phosphorylation of STAT-3, and activation of nuclear factor-kappaB (Maussang et al. 2009). High COX-2 and 5-LO expression and production of inflammatory mediators will also attract inflammatory cells capable of releasing inflammatory factors and reactive oxygen species causing inflammation, oxidative DNA damage, and impaired DNA repair mechanisms (Coussens and Werb 2002). In a mouse model with targeted expression of US28 in colon, mice developed inflammation associated adenocarcinoma (Bongers et al. 2010). Consistent with an important role for the virus to induce COX-2 expression, COX-2 inhibition obstructs HCMV replication (Zhu et al. 2002) and reduces growth of HCMV positive tumors in animal models (Baryawno et al. 2011; Maussang et al. 2009). In addition, combined antiviral therapy targeting HCMV (Valganciclovir) and COX-2 (Celecoxib) prevents HCMV replication and PGE2 production, further reduced meduloblastoma and neuroblastoma cell proliferation in vitro (Baryawno et al. 2011; Wolmer-Solberg et al. 2013) and tumor size in meduloblastoma xenografts (Baryawno et al. 2011).

Taken together, these observations raise the question of a possible impact of HCMV on tumor progression through induced production of potent inflammatory factors such as COX-2 and 5-LO that through their effects on both tumor cells and the tumor microenvironment may enhance tumor malignancy grade and promote tumor progression.

Epidemiological studies in large patient cohorts have reported that the cancer risk and metastasis development is reduced in patients who are treated with non-selective COX inhibitors (Anderson et al. 2002; Burn et al. 2011; Xu 2002). The critical role of inflammatory COX-2 and 5-LO and their biological products, prostaglandin and leukotrienes in inflammation and their ability to stimulate signalling pathways contributing to angiogenesis, tumor growth, and invasiveness, further highlights them as potential targets for cancer therapy. Notably, inhibitors of 5-LO were efficiently used to block proliferation of breast cancer cells and a lipoxygenase inhibitor administrated to one breast cancer patient successfully reverted multiple brain metastasis (Flavin 2007; Hammamieh et al. 2007; Poeckel et al. 2006). In vitro experiments and animal models using specific COX-2 inhibitors show a reduction in proliferation and invasion of breast cancer cells and an effect on tumor development and growth (Bocca et al. 2011; Na et al. 2013; Silva et al. 2012). Consistent with these findings, specific or non-specific inhibition of COX-2 has successfully been used for prevention and therapy of breast cancer (Arun and Goss 2004). Selective COX-2 inhibitors appear to be more protective but in both cases, with a significant reduction in the breast cancer risk (Ashok et al. 2011). However, the inhibition of both COX-2 and 5-LO would have additive benefits by simultaneously targeting both pathways of arachidonic acid metabolism. Indeed, combined treatment with COX-2 and 5-LO inhibitors showed a stronger effect on tumor cell proliferation and induction of apoptosis in colon cancer cells (Che et al. 2016; Cianchi et al. 2006). However, these compounds may also involve a so far not considered anti-viral effect against HCMV to affect tumor growth (Schroer and Shenk 2008; Zhu et al. 2002). As selective COX-2 inhibitors may confer increased risk of cardiovascular diseases and stroke (Trelle et al. 2011; Back et al. 2012; Martinez-Gonzalez and Badimon 2007; Sibbald 2004), there is a need to develop a non-toxic drug with potential to inhibit COX-2 and 5-LO, with therapeutic action in human cancers (Gautam et al. 2017).

In conclusion, we detected higher grades of HCMV-IE, COX-2 and 5-LO in the majority of BC samples than in adjacent non-malignant tissue specimens and a significant correlation between extensive HCMV-IE, COX-2 and 5-LO protein levels in infiltrating BCs. These results suggest that inflammation driven by COX-2 and 5-LO in human BC might be induced by HCMV in some patients and promote tumor progression. Thus, we suggest that anti-viral therapy targeting HCMV and inhibitors targeting COX-2 and 5-LO should be evaluated as new therapeutic options in selected breast cancer patients.

## Electronic supplementary material

Below is the link to the electronic supplementary material.

Supplemental Fig. 1A. Expression of HCMV-IE protein in HCMV infected placenta using IHC staining. Detection of HCMV-IE protein in HCMV infected placenta tissue section with IHC served as positive control. Omitting primary antibody in the staining protocol served as negative control. (TIFF 3372 kb)

Supplemental Fig. 1B. Expression of HCMV-IE protein could not be detected in breast cancer tissue using IHC staining. This HCMV negative breast tumor tissue section served as negative control for IHC staining used in this study. Staining for cytokeratin 5,6,8,17,19 served as positive control and cytokeratin 20 served as negative control in the staining protocol. (TIFF 1679 kb)

Supplemental Fig. 2A, B. No association was observed between COX-2 expression levels in BC tissues and increasing number of involved lymph node from N0 to N1 and N2 (A). No association was found between HCMV-IE and lymph node involvement (B). (TIFF 479 kb)

Supplemental Fig. 2C, D. No association was found between HCMV-IE or 5-LO and lymph node involvement or between HCMV-IE, COX-2, 5-LO and tumor size, and Ki-67 index (C, D). (TIFF 409 kb)

Supplemental Fig. 3A-C. No association was found between HCMV-IE, COX-2, 5-LO, and tumor size (A-C). (TIFF 606 kb)

Supplemental Fig. 3D-E. No association was found between HCMV-IE, COX-2, 5-LO, and KI-67 index (D-E). (TIFF 509 kb)
