# Supplementary figures and images for: Human cytomegalovirus infection is correlated with enhanced cyclooxygenase-2 and 5-lipoxygenase protein expression in breast cancer
Source: J Cancer Res Clin Oncol. 2019 Jun 15;145(8):2083–95. doi: 10.1007/s00432-019-02946-8 (PMC6658585; doi:10.1007/s00432-019-02946-8)

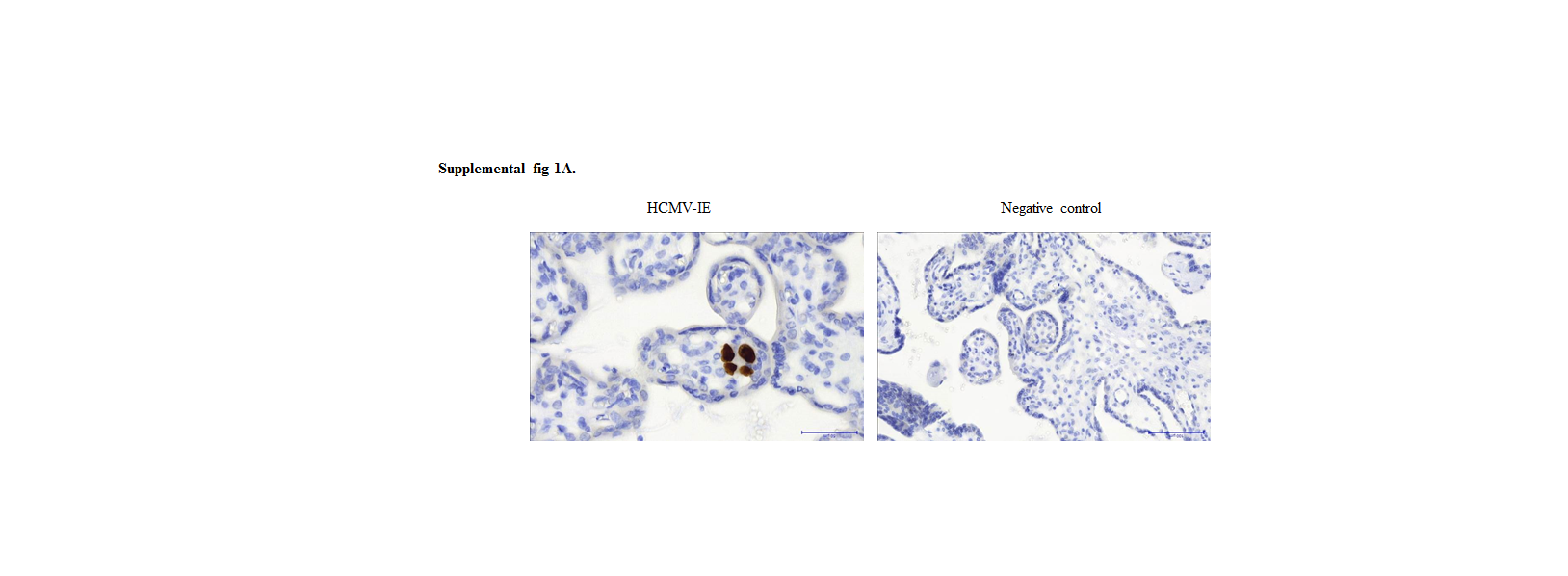

Supplement: Supplementary file 1 — Supplemental Fig. 1A. Expression of HCMV-IE protein in HCMV infected placenta using IHC staining. Detection of HCMV-IE protein in HCMV infected placenta tissue section with IHC served as positive control. Omitting primary antibody in the staining protocol served as negative control. (TIFF 3372 kb) [file 432_2019_2946_MOESM1_ESM.tif]

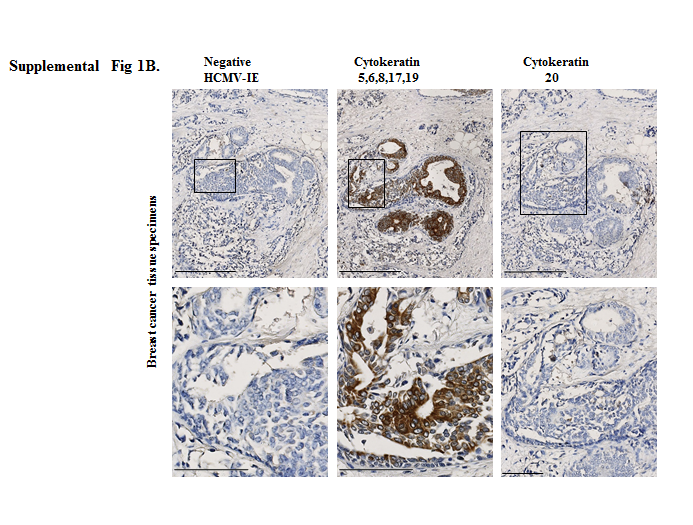

Supplement: Supplementary file 2 — Supplemental Fig. 1B. Expression of HCMV-IE protein could not be detected in breast cancer tissue using IHC staining. This HCMV negative breast tumor tissue section served as negative control for IHC staining used in this study. Staining for cytokeratin 5,6,8,17,19 served as positive control and cytokeratin 20 served as negative control in the staining protocol. (TIFF 1679 kb) [file 432_2019_2946_MOESM2_ESM.tif]

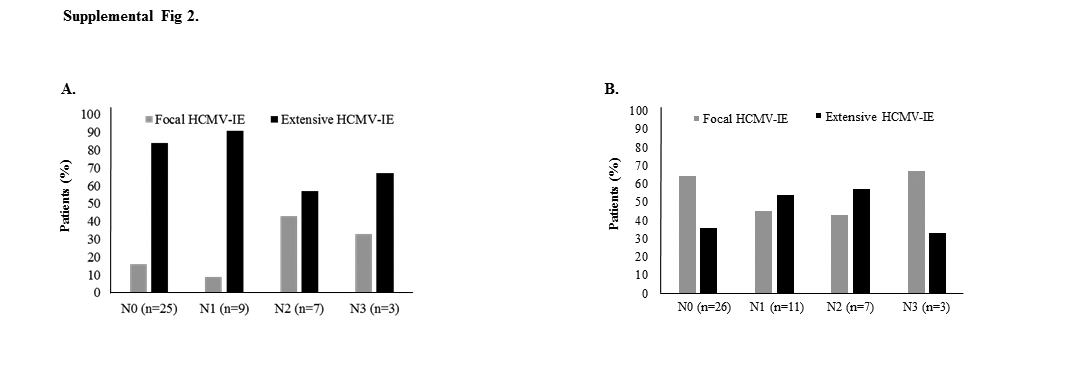

Supplement: Supplementary file 3 — Supplemental Fig. 2A, B. No association was observed between COX-2 expression levels in BC tissues and increasing number of involved lymph node from N0 to N1 and N2 (A). No association was found between HCMV-IE and lymph node involvement (B). (TIFF 479 kb) [file 432_2019_2946_MOESM3_ESM.tif]

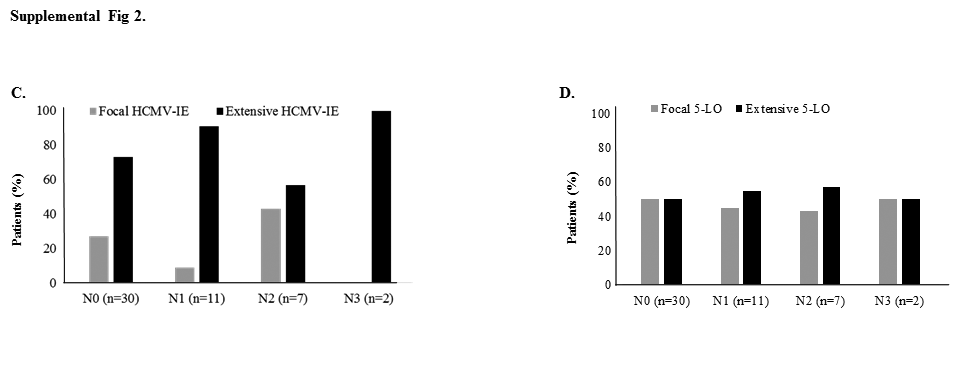

Supplement: Supplementary file 4 — Supplemental Fig. 2C, D. No association was found between HCMV-IE or 5-LO and lymph node involvement or between HCMV-IE, COX-2, 5-LO and tumor size, and Ki-67 index (C, D). (TIFF 409 kb) [file 432_2019_2946_MOESM4_ESM.tif]

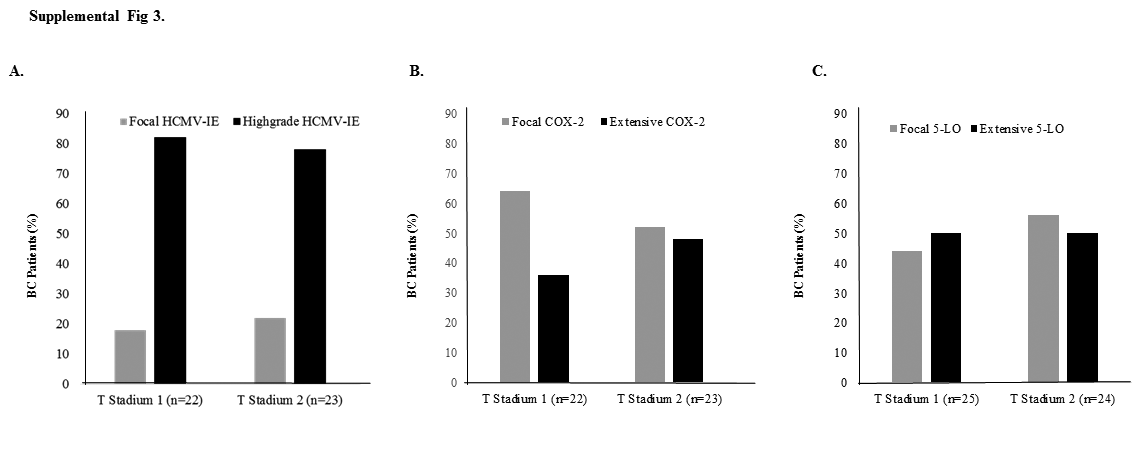

Supplement: Supplementary file 5 — Supplemental Fig. 3A-C. No association was found between HCMV-IE, COX-2, 5-LO, and tumor size (A-C). (TIFF 606 kb) [file 432_2019_2946_MOESM5_ESM.tif]

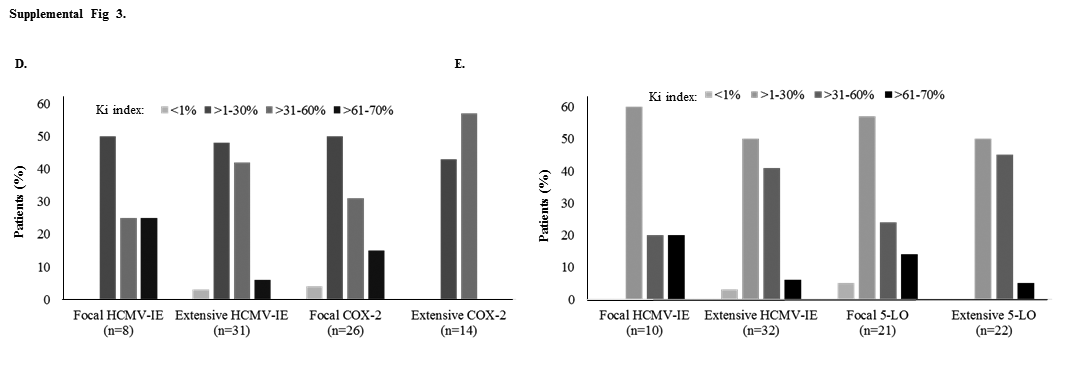

Supplement: Supplementary file 6 — Supplemental Fig. 3D-E. No association was found between HCMV-IE, COX-2, 5-LO, and KI-67 index (D-E). (TIFF 509 kb) [file 432_2019_2946_MOESM6_ESM.tif]
